# ^18^F-Labeling Using Click Cycloadditions

**DOI:** 10.1155/2014/361329

**Published:** 2014-05-27

**Authors:** Kathrin Kettenbach, Hanno Schieferstein, Tobias L. Ross

**Affiliations:** ^1^Institute of Nuclear Chemistry, Johannes Gutenberg University Mainz, 55128 Mainz, Germany; ^2^Radiopharmaceutical Chemistry, Department of Nuclear Medicine, Hannover Medical School, 30625 Hannover, Germany

## Abstract

Due to expanding applications of positron emission tomography (PET) there is a demand for developing new techniques to introduce fluorine-18 (*t*_1/2_ = 109.8 min). Considering that most novel PET tracers are sensitive biomolecules and that direct introduction of fluorine-18 often needs harsh conditions, the insertion of ^18^F in those molecules poses an exceeding challenge. Two major challenges during ^18^F-labeling are a regioselective introduction and a fast and high yielding way under mild conditions. Furthermore, attention has to be paid to functionalities, which are usually present in complex structures of the target molecule. The Cu-catalyzed azide-alkyne cycloaddition (CuAAC) and several copper-free click reactions represent such methods for radiolabeling of sensitive molecules under the above-mentioned criteria. This minireview will provide a quick overview about the development of novel ^18^F-labeled prosthetic groups for click cycloadditions and will summarize recent trends in copper-catalyzed and copper-free click ^18^F-cycloadditions.

## 1. Introduction

For the application in positron emission tomography (PET) [1], fluorine-18 provides ideal nuclear physical characteristics for* in vivo* imaging. Fluorine-18 offers a half-life of 110 min, a *β* ^+^-branch of 97%, and especially a low *β* ^+^-energy of 635 keV, which is responsible for a very high spatial resolution [2]. The challenges for researchers are to develop convenient ^18^F-labeling strategies, which include short reaction times and applicability for sensitive biomolecules. Especially the harsh conditions during direct ^18^F-labeling pose an exceeding challenge [3, 4]. Therefore, most of the radiolabeling strategies focus on ^18^F-containing prosthetic groups, which allow a sensitive and bioorthogonal ^18^F-labeling to treat the multitude of functional groups in those bioactive compounds with respect.

The most established method, which fulfills all mentioned criteria, is given by click reactions. Especially the Cu(I)-catalyzed variant of the Huisgen 1,3-dipolar cycloaddition of terminal alkynes and azides offers a very powerful reaction with high specificity and excellent yields under mild conditions [5]. As a result, numerous PET tracers have been synthesized using CuAAC in a widespread spectrum of structural varieties of the prosthetic group within the last decade. One of the latest investigations deals with a polar clickable amino acid-based prosthetic group to further improve the pharmacokinetic properties of radiotracers, particularly suitable for peptides and proteins [6].

However, the need of cytotoxic copper during CuAAC has led to the necessity of alternative fast and copper-free click reaction strategies for radiofluorination and additionally enabling pretargeting approaches in living systems. Those so-called strain-promoted click reactions can be carried out between cyclooctyne derivatives and azides (strain-promoted azide-alkyne cycloaddition, SPAAC) [7–13] or tetrazines (tetrazine-trans-cyclooctyne (TTCO) ligation) [14–17] as well as between norbornene derivatives and tetrazines [18]. Especially, the TTCO ligation showed promising reaction rates, which makes this click reaction concept very suitable for ^18^F-labeling and also for* in vivo* application in living systems. Very recently, new versions of ^18^F-click cycloadditions are added to the range of reactions [19–25]. In this line, the first ^18^F-labeled *β*-lactame became available via a new* radio*-Kinugasa reaction [21].

As a consequence, click cycloaddition is one of the most frequently applied methods for ^18^F-labeling of new bioactive compounds, with or without a catalytic system. This can be impressively illustrated by the fact that over 50 original papers have been published in this research area within the last eight years.

**Figure 1:**
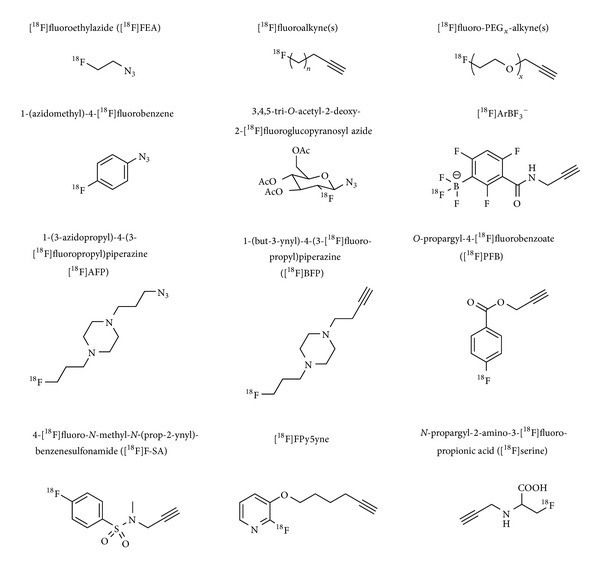
Lead structures of the most important ^18^F-prosthetic groups applied for copper-catalyzed click ^18^F-fluorination.

Tables 1–3 give an overview of the ^18^F-prosthetic groups, the reaction conditions and reaction partners applied for copper-catalyzed, copper-free and other kinds of ^18^F-click cycloadditions, respectively. The most important structures of those prosthetic groups are shown in Figures 1, 3, and 5.

## 2. Copper-Catalyzed ^**18**^ **F**-Click Cycloadditions

In the last decade, the copper-catalyzed azide alkyne cycloaddition (CuAAC), which has first been reported independently by Rostovtsev et al. [81] and Tornøe et al. [82] in 2002, has spread over almost all fields of chemistry [83–87], biology [88–90], and material science [91, 92]. The great advantage of this method is given by its outstanding efficiency, its regiospecificity, and fast formation of 1,4-disubstituted 1,2,3-triazoles at ambient temperatures, which is particularly suitable for ^18^F-labeling of sensitive biomolecules. In particular, the CuAAC enables incorporation of fluorine-18 via a prosthetic group under mild and bioorthogonal conditions [22–25]. 1,2,3-triazoles were first introduced by Michael, who described the formation of a 1,2,3-triazole from a phenylazide in 1893 [93]. Following this pioneering work, Dimroth, Fester, and Huisgen described this type of reaction as a 1,3-dipolar cycloaddition for the first time in 1963 [5].

In 2006, Marik and Sutcliffe published the application of the CuAAC as an ^18^F-labeling strategy for the first time [26]. They radiolabeled three different alkyne precursors in radiochemical yields (RCY) of 36–81%. Afterwards they were reacted them with azido-functionalized peptides in RCY of 54–99% and an overall reaction time of 30 min. Thus, they could show a new, very fast, efficient, and mild ^18^F-labeling strategy for complex compounds, especially appropriate for sensitive biomolecules. Only two years later, the suitability of this approach was demonstrated for the ^18^F-labeling of a folate derivative for* in vivo* tumor imaging with the same prosthetic group, 6-[^18^F]fluoro-1-hexyne [30]. The radiofolate was obtained in RCY of 25–35% and was applied to KB-tumor bearing mice. A specific tumor accumulation could be observed by using the folate receptor (FR) targeting concept. Furthermore, Kim et al. used ^18^F-labeled alkynes as prosthetic groups for the ^18^F-labeling of 2,3,4,6-tetra-O-acetyl-*β*-D-glucopyranosyl azide [27], which in turn was employed to label the *α*_V_ *β*_6_ specific peptide A20FMDV2 [28].

Considering all known clickable prosthetic groups for ^18^F-labeling, [^18^F]fluoroethyl azide ([^18^F]FEA) is certainly one of the most investigated clickable ^18^F-prosthetic groups. Until today, about twenty different manuscripts deal with [^18^F]FEA to radiolabel a broad variety of biomolecules and compounds. In 2007, Glaser and Årstad [31] mentioned for the first time the preparation of [^18^F]FEA with a RCY of 55% using 2-azidoethyl-4-toluenesulfonate as precursor. As a proof of concept, they reacted [^18^F]FEA with different terminal alkynes in very good to excellent RCY of 61–98%. With respect to the catalytic system copper sulfate in combination with ascorbic acid or sodium ascorbate has mainly been used, whereas only in a few approaches copper(I) iodide was used [37, 42]. It has been shown that addition of bathophenanthroline disulfonate (Cu^I^ stabilizing agent) accelerates the 1,3-dipolar cycloaddition [36, 38, 45]. The very good access to [^18^F]FEA led to the development of a variety of radiotracers labeled with this prosthetic group, like ^18^F-deoxyuridine [37], ^18^F-fluoro-oxothymidine (^18^F-FOT), or ^18^F-fluoro-thiothymidine (^18^F-FTT) [40] as well as apoptosis markers [36] and several peptide systems [34, 44, 49]. In 2012, Smith et al. [40] described the reduction of [^18^F]FEA using copper wire under acidic conditions, which is a possible explanation of the poor yields during some click reactions.

In 2007, Sirion et al. [50] reported for the first time [^18^F]fluoro-PEG_*x*_-derivatives (*x* = various polyethylene glycol (PEG) ratios) as new ^18^F-labeled prosthetic click groups. These compounds showed a reduced volatility and increased polarity compared with other ^18^F-labeled prosthetic groups like [^18^F]FEA or [^18^F]fluoroalkynes. These properties ease their handling as well as improving the* in vivo* behavior of the labeled compounds. The compounds showed a longer circulation time and a reduced renal clearance making them very suitable for* in vivo* application. Sirion et al. described the preparation of different aliphatic and aromatic ^18^F-PEG-azides and ^18^F-labeled alkynes in RCY of 85–94%. As a proof of concept, they carried out cycloadditions with the ^18^F-labeled prosthetic groups and the corresponding alkynes, respectively, azides in high RCY of 71–99%. Several other groups continued this work by using the ^18^F-labeled PEGylated prosthetic groups for labeling cRGD derivatives [51] and other peptides [53], nanoparticles [52, 54], or folates [55].

To increase the lipophilicity and metabolic stability of radiotracers, [^18^F]fluoro-aryl-based prosthetic groups have been developed and investigated. In 2007, Ramenda et al. [56] published for the first time a 4-[^18^F]fluoro-*N*-methyl-*N*-(prop-2-ynyl)-benzenesulfonamide (p-[^18^F]F-SA), which was obtained in RCY of 32 ± 5%. Subsequently, this prosthetic group was used for radiolabeling an azido-functionalized neurotensin giving a RCY of 66%. Furthermore, the same group used the ^18^F-aryl prosthetic group for the labeling of human serum albumin (HSA) [57] and other proteins, phosphopeptides, and* L*-RNA [58] in good RCY. A pyridine-based ^18^F-prosthetic group was first introduced by Inkster et al. [59] in 2008 by reacting [^18^F]FPy5yne with a model peptide in RCY of 18.7% and an overall reaction time of 160 min. They started from either 2-nitro- or 2-trimethylammonium pyridine to synthesize [^18^F]FPy5yne with a RCY of 42%. Furthermore, [^18^F]pyridine derivatives have been used to radiolabel cRGDs [60] and the* D*-amino acid analog of WT-pHLIP [61].

In 2009, Vaidyanathan et al. [62] presented a prosthetic group based on a 4-[^18^F]fluorobenzoate. Propargyl-4-[^18^F]fluorobenzoate ([^18^F]PFB), which could be obtained in RCY of 58 ± 31% within 15 min. To investigate the labeling properties of this new prosthetic group, numerous compounds have been ^18^F-labeled using [^18^F]PFB with RCY from 37% to 88% and overall reaction times of about 1 h. Another approach was published by Li et al. in 2012 [63], who synthesized 4-[^18^F]fluoro-3-nitro-*N*-2-propyn-1-yl-benzamide ([^18^F]FNPB) for ^18^F-labeling of cRGDfK and a D4 peptide, which was identified as an EGFR targeting ligand. This approach was followed by the synthesis of 1-(azidomethyl)-4-[^18^F]fluorobenzene by Thonon et al. [64]. They did a multistep radiosynthesis (4 steps), where the fluorine-18 was introduced in the first step. The desired radiolabeled product could be obtained in a RCY of 34% within 75 min and was used itself to label a 4-ethynyl-*L*-phenylalanine-containing peptide. The same prosthetic group was also employed by Mercier et al. [65] and Flagothier et al. [66] for ^18^F-labeling of* si*RNA. Other structural analog prosthetic groups have also been developed by Mercier et al. [65] and Chun and Pike [67].

To improve the* in vivo *behavior of peptides with respect to blood clearance and stability, Maschauer and Prante developed ^18^F-gluco-derivatives for CuAAC-radiolabeling of Fmoc-*L*-propargylglycine with a RCY of 60% [68]. They showed that the ^18^F-click labeling reaction was more convenient by using the *β*-anomeric derivative of the azides, respectively, alkynes, giving very high RCY of 71 ± 10%. One year later, they published the first* in vivo* evaluation of an ^18^F-labeled RGD peptide labeled with [^18^F]FDG-*β*-Az in U87MG-tumor bearing mice showing an improved blood clearance and stability [65, 66]. Likewise, Fischer et al. demonstrated in 2012 that a [^18^F]fluorodeoxyglycosyl folate could be obtained in RCY of 5–25% and subsequent biodistribution and PET-imaging studies showed a high and specific uptake of the radiotracer in FR-positive tumors [70]. The variety of new ^18^F-labeling strategies using ^18^F-Fluoroglycosylation is the focus of a review article as a part of this special issue provided by Maschauer and Prante [94].

As another promising approach, Li et al. presented in 2013 an alkyne-functionalized aryltri-[^18^F]fluoroborate for radiolabeling azido-bombesin and azido-RGD. The major advantage of this method is the two-step, one-pot procedure providing a water-soluble and noncoordinating aryltri-[^18^F]fluoroborate anion, which provided specific activities up to 555 GBq/*μ*mol [75, 76, 95].

Two new piperazine-based prosthetic groups, 1-(but-3-ynyl)-4-(3-[^18^F]fluoropropyl)piperazine ([^18^F]BFP) and 1-(3-azidopropyl)-4-(3-[^18^F]fluoropropyl)piperazine ([^18^F]AFP), have recently been developed by Pretze and Mamat [78]. Spiro salts were used as precursors, facilitating purification by using solid phase extractions (RP-18 or SiO_2_-cartridges). Both prosthetic groups could be obtained in RCY of about 30% using an automated synthesis module. To avoid Glaser coupling, which has been observed by using [^18^F]BFP for radiolabeling of peptides, [^18^F]AFP was used instead. An important observation was the fact that the applied peptide formed very strong complexes with the copper catalyst, which required the use of bispidine as a strong chelating agent to remove cytotoxic copper species.

**Figure 2:**
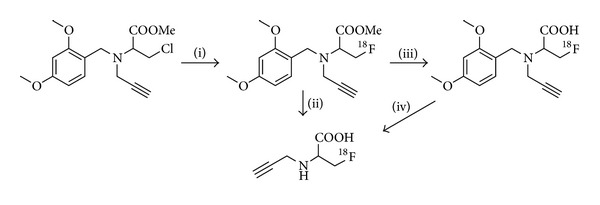
Radiosynthesis of a new amino-acid based ^18^F-prosthetic group (*N*-propargyl-2-amino-3-[^18^F]fluoro-propionic acid, “[^18^F]serine”) for ^18^F-CuAAC-labeling of complex biomolecules. (i) [K ⊂ 2.2.2]^+^/^18^F^−^, DMSO, 140°C, 10 min; (ii) hydrochloric acid (3.3 M), 100°C, 15 min; for analytical purposes (sequential deprotection): (iii) sodium hydroxide (3.3 M), 60°C, 5 min; (iv) hydrochloric acid (3.3 M), 100°C, 15 min.

**Figure 3:**
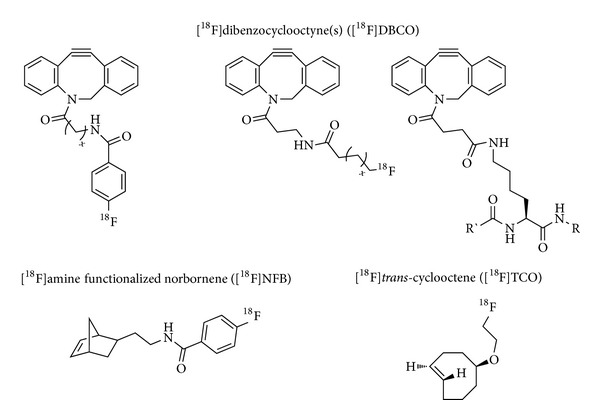
Lead structures of the most important ^18^F-prosthetic groups applied for copper-free click ^18^F-fluorination.

One of the latest developments describes the synthesis of an ^18^F-labeled alanine derivative as a new prosthetic click group, reported by Schieferstein and Ross [6]. In this case, an amino acid-based prosthetic group has been developed to improve the pharmacokinetic profile of ^18^F-click-labeled biomolecules. The prosthetic group was obtained in good RCY of 28 ± 5% from a two-step reaction as described in Figure 2. The final ^18^F-labeled prosthetic group was subsequently reacted with an azido-RGD as model system in RCY of 75% within 20 min.

Considering the above-mentioned prosthetic groups for radiolabeling with fluorine-18, Table 1 summarizes important properties of those components. It has been shown that the integration of an ^18^F-propyl, ^18^F-ethyl, or ^18^F-aryl moiety can provide an improved metabolic profile and that the glycosylation or PEGylation can further improve the* in vivo* behavior. Furthermore, for* in vivo* application a total removal of the copper catalyst is essential. This could be very challenging in the case where peptides or proteins are able to complex copper species from the catalytic system.

## 3. Copper-Free ^**18**^F-Click Cycloadditions

Even though a large number of novel radiotracers using click chemistry have been developed, none of them has entered clinical routine to date, apart from ^18^F-RGD-K5, which is already used in clinical trials in US. This can be explained by the need of cytotoxic copper during radiotracer syntheses by using copper-catalyzed 1,3-dipolar Huisgen cycloadditions [96]. Thus, there is still a demand for facile (metal-free) and robust ^18^F-labeling reactions for the syntheses of radiotracers for imaging of malignancies* in vivo*. This leads to the development of catalyst-free click-labeling approaches, which spare copper species during labeling steps and even enable* in vivo* pretargeting concept. Recent developments deal with biocompatible strain-promoted copper-free versions of the alkyne-azide cycloaddition (SPAAC), where the focus has been set on derivatives of cyclooctynes and dibenzocyclooctynes. First approaches focus on the reaction of ^18^F-labeled cyclooctynes with azide-bearing biomolecules. On the other hand, in further approaches cyclooctyne-carrying bioactive compounds are used, which can be labeled with different ^18^F-labeled azides. In the beginning, only a few studies have been reported due to the complex and low yielding syntheses of strained cyclooctynes [10, 12, 14]. However, nowadays lots of cyclooctyne derivatives are commercially available, which facilitates the precursor syntheses and opens a wide range of applications.

In 2011 Bouvet et al. [7] published the first example of a SPAAC with ^18^F-labeled* aza*-dibenzocyclooctyne, [^18^F]FB-DBCO, and a plethora of azides. The ^18^F-labeled building block was synthesized via acylation of commercially available* N*-(3-aminopropionyl)-5,6-dihydro-11,12-didehydrodibenzo[*b,f*]azocine with* N*-succinimidyl-4-[^18^F]fluorobenzoate ([^18^F]SFB), which can be easily prepared in an automated synthesis module [97]. The ^18^F-labeled cyclooctyne could be obtained in a RCY of 85% and a purity >95% within 60 min. The evaluation of this building block in healthy Balb/C mice showed 60% of intact compound at 60 min p.i. and had a blood clearance half-life of 53 s. Besides, the compound was stable in methanol and phosphate buffer over 60 min. Subsequently, [^18^F]FB-DBCO was reacted with various azides as proof of principle showing different structural complexities. In all reactions, the formation of two regioisomers (1,4- and 1,5-triazole) has been observed and in some cases a separation of the regioisomers by HPLC was impossible. All ^18^F-labeled radiotracers were obtained in good to excellent RCY of 69–98% within an overall reaction time of about 2 h. However, the reaction rates in these cases were much slower compared to other examples of bioorthogonal reactions, limiting this new approach for* in vivo* pretargeting applications.

A cyclooctyne derivative has been conjugated to bombesin (*aza*-DBCO-BN, 9 steps) with an overall yield of 17% by Campbell-Verduyn et al.   [8]. The* aza*-DBCO-BN was reacted with various ^18^F-azides giving RCY of 19–37% within 30 min. In 2011, Arumugam et al. [9] investigated the direct ^18^F-labeling of azadibenzocyclooctyne (DBCO) yielding the ^18^F-labeled prosthetic group (RCY = 36%). The radiolabeling was followed by a click reaction with an* azido*-octreotide leading to the ^18^F-labeled octreotide in a RCY of 95% within a total reaction time of 1.5 h. In contrast, other working groups used ^18^F-cyclooctynes for labeling RDG-derivatives [11] as well as further integrin-specific peptides [10, 13].

**Figure 4:**
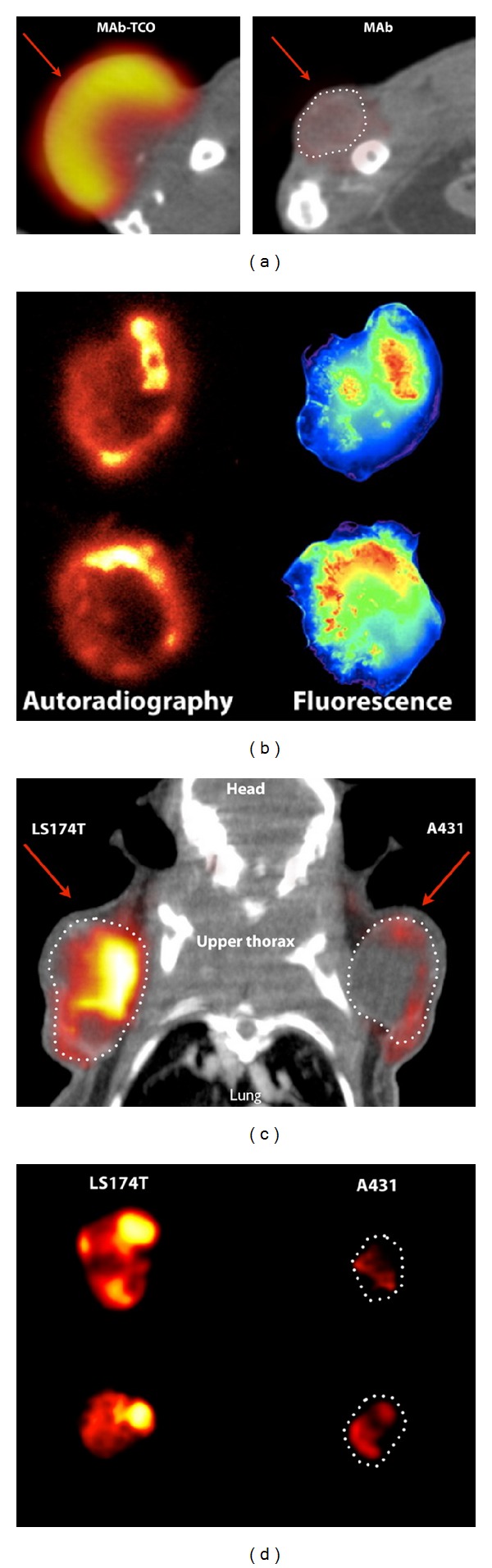
PET and autoradiography using ^18^F-tetrazine agents. (a) PET/CT fusion of LS174T tumor xenograft labeled using either* trans-*cyclooctene (TCO) monoclonal antibodies (mAb TCO) or control unlabeled antibodies (mAb) followed by ^18^F-PMT10 (polymer-modified tetrazine). Arrows indicate location of the tumor xenograft. The bladder was omitted for clarity. (b) Imaging using autoradiography (left side) and fluorescence slices after targeting with fluorescence TCO monoclonal antibody and ^18^F-PMT10. (c) PET/CT fusion of mouse bearing A431 and LS174T tumors after targeting with anti-A33 TCO monoclonal antibodies followed by ^18^F-PMT10. Arrows indicate location of tumors and the liver was omitted for clarity. (d) Autoradiography of representative 1 mm LS174T and A431 tumor slices after multistep targeting (reprinted with permission from [80]; Copyright 2012 National Academy of Sciences of the United States of America).

**Figure 5:**
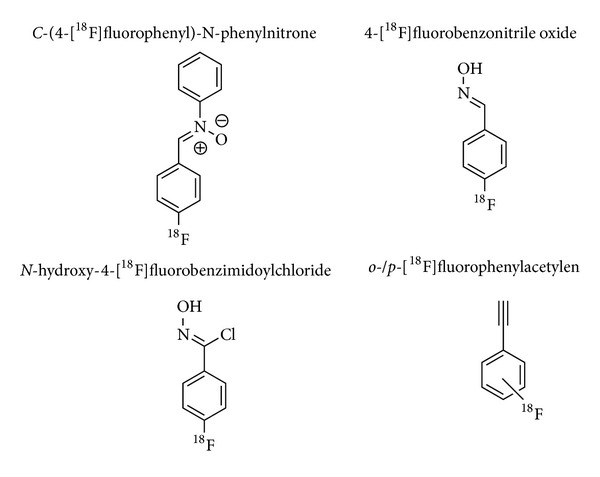
Lead structures of new ^18^F-prosthetic groups applied for click ^18^F-fluorination.

Another possibility to perform copper-free click reactions is given by the inverse electron demand of the Diels Alder cycloaddition between a cyclooctene and a tetrazine under the release of nitrogen. The so-called tetrazine-*trans*-cyclooctene ligation (TTCO ligation) was first published by Li et al. in 2010 [14]. Concerning the instability of the tetrazines, it is more practical to functionalize the biomolecule with a tetrazine followed by the reaction with an ^18^F-labeled cyclooctene. The latter are much more suitable for direct ^18^F-labeling than tetrazines. For this purpose a nosylate precursor was used for ^18^F-labeling of the cyclooctene providing RCY of 71% within 15 min. To investigate the suitability of the ^18^F-prosthetic group in click reactions, the ^18^F-cyclooctene was reacted with a 3,6-di(2-pyridyl)-*S*-tetrazine in an excellent RCY of 98% within 10 s, showing its outstanding feasibility for* in vivo* pretargeting approaches. These fast reaction rates made this approach very attractive that even ^11^C-labeling reaction was explored using the inverse electron demand Diels Alder cycloaddition between a cyclooctene and a tetrazine [98]. In 2011, ^18^F-labeled cyclooctene was linked to a tetrazine-RGD derivative by Selvaraj et al. [15] with a RCY of 90% within 5 min at room temperature. The resulting ^18^F-labeled tracer was tested in* in vivo* experiments showing a high tumor accumulation, which could selectively be blocked. In 2012, the group of Devaraj et al. [80] published for the first time the* in vivo* click reaction of [^18^F]*trans*-cyclooctene and a polymer-modified tetrazine (PMT). The radiolabeled peptide ^18^F-PMT10 could be obtained in a RCY of 89.2%. Whole body animal PET scans were carried out 3 h p.i., showing renal clearance and a widespread tissue distribution as can be seen in Figure 4. Previously, the same group described the synthesis of an ^18^F-labeled cyclooctene with a RCY of 46.1 ± 12.2%. Subsequently, this prosthetic group was clicked with a tetrazine-modified exendin-4 in RCY of 46.7 ± 17.3% [16].

A similar strategy was published by Knight et al. in 2013, where an ^18^F-labeled amino-functionalized norbornene was reacted with a tetrazine-modified peptide [18]. The ^18^F-labeled norbornene was obtained using N-succinimicyl-4-[^18^F]fluorobenzoate ([^18^F]SFB) in RCY of 60 ± 17% within 52 min. As a proof of concept, two different tetrazines, an asymmetric dipyridyl tetrazine, and a tetrazine-modified bombesin peptide were labeled with ^18^F-labeled norbornene derivative ([^18^F]NFB) in 46–97% RCY within 82 min.

Considering the copper-free click labeling of bioactive compounds with fluorine-18, both the strain-promoted alkyne-azide cycloaddition (SPAAC) and the tetrazine-*trans*-cyclooctyne ligation (TTCO ligation) show promising results. Regarding* in vivo* pretargeting approaches, only the TTCO ligation showed favorable results and reaction rates, which are suitable for this application [80].  Table 2 summarizes reaction conditions, radiochemical yields, and reaction partners of those components.

## 4. New Developments in ^**18**^F-Click Cycloadditions

The latest developments in metal-free ^18^F-click cycloadditions have been reported by Zlatopolskiy et al. [19–21] (Table 3). In a first approach, the ^18^F-labeled building block C-(4-[^18^F]fluorophenyl)-N-phenyl nitrone was developed to form ^18^F-isoxazolidines via high-yielding [3+2]cycloadditions with various maleimides [19]. C-(4-[^18^F]fluorophenyl)-N-phenyl nitrone was obtained from the reaction of 4-[^18^F]fluorobenzaldehyde and N-phenylhydroxylamine in high RCY of 74% with 10 min. In the subsequent click cycloaddition step, differently substituted maleimides as model dipolarophiles were used to form the corresponding isoxazolidines as endo-/exoisomers in high yields of up to >90% within 10 min. A one-pot strategy with* in situ* generation of C-(4-[^18^F]fluorophenyl)-N-phenyl nitrone provided the desired ^18^F-isoxazolidines only in moderate yields of 25% and only after heating to 110°C. Under optimized conditions, ^18^F-isoxazolidines were obtained from fast ^18^F-click [3+2]cycloadditions.

In further studies, the same group used 4-[^18^F]fluorobenzonitrile oxide instead of C-(4-[^18^F]fluorophenyl)-N-phenyl nitrone as 1,3-dipol for milder reaction conditions [20] (Table 3). 4-[^18^F]fluorobenzonitrile oxide was obtained in 92% RCY within 10 min from the reaction of 4-[^18^F]fluorobenzaldehyde (RCY: 30–50%, 50 min [99]) with hydroxylamine and subsequent treatment with phenyl iodine bis(trifluoroacetate).

After the click [3+2]cycloaddition to various ^18^F-labeled model 2-isoxazolines and isoxazoles was successfully tested, the novel method was applied to three different COX-2 inhibitors (indomethacin conjugates) carrying dipolarophilic moieties of cyclononyne, maleimide, and propyne. The resulting products were obtained in moderate to excellent RCY of 81%, 55%, and 35%, respectively. It is noteworthy that, for the propyne derivative, the milder oxidant [bis(acetoxy)iodo]benzene was used to avoid decomposition. Finally, the method was successfully adapted for ^18^F-labeling of two model dipeptide conjugates, cyclononyne- and norbornene-*β*-Ala-Phe-OMe. However, the original cycloaddition using 4-[^18^F]fluorobenzonitrile oxide did only provide traces of the desired products. Consequently, 4-[^18^F]fluorobenzonitrile oxide was further treated with chloramine T (CAT)* in situ* forming the more stable building block N-hydroxy-4-[^18^F]fluorobenzimidoyl chloride. With the use of high precursor (peptides) amounts, the latter enabled excellent RCY of the ^18^F-labeled dipeptides of up to 88% within 10 min at room temperature [20]. Under optimized conditions low precursor amounts of 5 nmol (cyclononyne) and 50 nmol (norbornene-*β*-Ala-Phe-OMe) still allowed RCY of 56% and 47%, respectively.

In a very recent report, Zlatopolskiy and coworkers applied their ^18^F-labeled nitrone, C-(4-[^18^F]fluorophenyl)-N-phenyl nitrone, for the first formation of ^18^F-labeled *β*-lactames via the CuI-catalyzed Kinugasa reaction [21] (Table 3). The optimized reactions went smooth under very mild conditions to give the ^18^F-labeled model *β*-lactames in high RCY and various isomeric mixtures of the* trans*- and* cis*-product. In dependency on the reactivity of the terminal alkynes, the reaction parameters needed (individual) optimization regarding catalyst system, solvent, temperature, and CuI-stabilizing ligands. As a biologically relevant molecule the ^18^Flabeled nucleobase chimera was synthesized as potential PET-imaging agent for bacterial infections.

Moreover, the dipeptide *β*-Ala-Phe-OMe was propiolated and used in this radio-Kinugasa reaction to give excellent RCY of 85% of the ^18^F-labeled dipeptide under very mild conditions (aqueous solution, room temperature) [21]. Similarly, this new method was successfully transferred to the ^18^F-labeling of proteins. Bovine serum albumin (BSA) was conjugated with 3-propiolamidopropyl chloroformate. This propiolated BSA was successfully radiolabeled with fluorine-18 in the radio-Kinugasa reaction.

## 5. Conclusions

The field of click cycloadditions had and still has a major impact in ^18^F-labeling chemistry. The very mild reaction conditions mostly applicable and the excellent efficiency of all types of these reactions are particularly suitable for ^18^F-labeling. Especially, complex and sensitive biomolecules benefit from this methodology. No protection group chemistry is needed and the ^18^F-click cycloaddition step provides the final radiotracer.

Besides several new ^18^F-labeled radiotracers are available via click cycloadditions, and the metal-free versions even enabled pretargeting concepts by* in vivo* click. The latest development of a radio-Kinugasa reaction towards the first ^18^F-*β*-lactames demonstrates the highly active field and the broad applicability of ^18^F-click cycloadditions.

## Figures and Tables

**Table 1:** Summary of the prosthetic groups, reaction conditions, and reaction partners applied for copper-catalyzed click ^18^F-fluorination.

^ 18^F-prosthetic group	Steps/reaction time^1^	RCY^2^	Reacting agent	Catalytic system	Overall reaction time^1^ (CCA)	RCY^2^ CCA	Literature
[^**18**^ **F**] **fluoroalkynes**	1 step, 10 min	36–81%	N-(3-azidopropionyl) peptides	CuI/NaAsc/DIPEA	30 min	54–99%	[26]
4-[^18^F]fluoro-1-butyne	1 step, 15 min (estimated)	n.d.	Glucopyranosyl azide		75–80 min	30%	[27]
4-[^18^F]Fluoro-1-butyne	1 step, 15 min	45 ± 3%	2,3,4,6-tetra-O-acetyl-b-D-glucopyranosyl azide	Cu(I)/Asc/2,6-lutidine	30 min	27 ± 6%	[28]
5-[^18^F]fluoro-1-pentyne	1 step, 15 min	59 ± 6%				52 ± 5%	
	1 step, 22 min	86 ± 2%	*α*_V_ *β*_6_ specific peptide A20FMDV2 azide	CuI/Asc	66 min	8.7 ± 2.3%	[29]
6-[^18^F]fluoro-1-hexyne	1 step, 12 min	70–85%	*γ*-(4-azido-butyl)-folic acid amide	CuI	1.5 h	25–35%	[30]

[^**18**^ **F] fluoroethyl azide** **([** ^**18**^ **F]** **FEA)**	1 step, 15 min	55%	Terminal alkynes	Excess of Cu^2+^/Asc or copper powder	1 h	61–98% respectively 15–98% with copper powder	[31] [32]
		n.d.	Caspase 3/7 Selective Isatin	CuSO_4_/Asc	n.d.	65 ± 6%	[33]
			RGD peptides	Cu^2+^/Asc		47 ± 8%	[34]
			3-Cyanoquinoline core		3 h	37 ± 3.6%	[35]
			Apoptosis marker ICMT11	CuSO_4_/Asc/BPDS	n.d.	1–3.4% n.d.c.	[36]
			5-Ethynyl-2′-deoxyuridine	CuI/ascorbic acid/DIPEA		75 ± 10%	[37]
			[Tyr^3^]octreotate analogues	CuSO_4_/Asc/BPDS	30 min (estimated)	40–64%	[38]
			ICMT-11 (automated synthesis)		90 min	3 ± 2.6% n.d.c.	[39]
			Nucleosides	CuSO_4_/Asc	n.d.	8–12% n.d.c.	[40]
			4-(prop-2-ynyloxy)Benzaldehyde		35 min	90%	[41]
			Haloethylsulfoxides	CuI/ascorbate/DIPEA	n.d.	28.5 ± 2.5%	[42]
		50% n.d.c.	Nitroaromatic substrates	CuSO_4_/Asc	1 h		[43]
		71 ± 4%	RGDfK		60 min	60 ± 2%	[44]
		55%	Alkyne-func. 6-halopurines	One-pot BPDS-copper(I) (CuSO_4_/NaAsc.)	1 h	55–75%	[45]
		n.d.	tert-butyl ester of N-Boc-(S)-propargyl glycine	CuSO_4_, NaAsc	2.5 h	58 ± 4%	[46]
	Precursor: 2 steps [^18^F]FEA: 15 min.	n.d.	3-Butynyl triphenyl phosphonium bromide		1 h	n.d.	[47]
	1 step, 5–10 min	68–75%	Alkynes of benzene rings		30 min	25–87%	[48]
[^18^F] FEA from a polyflourinated sulfonate precursor	n.d.	n.d.	FtRGD		70–75 min	10–30% n.d.c.	[49]

^** 18**^ **F-Fluoro-PEG-Alkyne**	1 step, 20 min	85–94%	Various azides	CuSO_4_/Asc	10–30 min	71–99%	[50]
	1 step, 15 min	65 ± 1.9%	E(RGDyK)_2_ azide		110 min (estimated)	52 ± 8.3%	[51]
		57%	Nanoparticle azide		1 h (estimated)	58%	[52]
[^18^F]PEG_3_-azide	1 step, 40 min	62 ± 4%	N-alkynylated peptide	CuSO_4_/Asc/BPDS	2 h (estimated)	31 ± 6%	[53]
		n.d.	ZnO nanoparticle alkynes		n.d.	>95%	[54]
[^18^F]PEG-azide	Precursor: 2 steps labeling: 1 step	labeling: 58%	*γ*-(11-azido-3,6,9-trioxaundecanyl)folic acid amide	CuAcetate, NaAsc	2.5 h	8.5%	[55]

**4-** **[^18^F]** **fluoro-N-methyl-N-(prop-2-ynyl)-** **benzenesulfonamide (p** **[^18^F]** **F-SA)**	Precursor: 3 steps, labeling: 1 step, 80 min	32 ± 5%	Azide-functionalized neurotensin	Cu(I)-TBTA	n.d.	66%	[56]
			Azide-functionalized human serum albumin (HSA)		100 min	55–60%	[57]
		n.d.	Azide-functionalized phosphopeptide, protein (HAS), oligonucleotide (L-RNA)	CuSO_4_/Asc	2 h	77%/55–60%/25%	[58]

**[^18^F]** **FPy5yne**	1 step, 15 min	42%	N_3_–(CH_2_)4–CO–YKRI–OH (BG142)	Tetrakis(acetonitrilo) copper(I) hexa fluorophosphates/TBTA	160 min	18.7%	[59]
			Azide-functionalized DNA	CuBr/TBTA and 2,6-lutidine	276 min	24.6 ± 0.5%	
2-[^18^F]fluoro-3-pent-4-yn-1-yloxypyridine ([^18^F]FPyKYNE)	20–25 min	20–35%	Azide-functionalized RGD peptide	CuSO_4_/Asc	125 min	12–18%	[60]
6-[^18^F]fluoro-2-etynylpyridine	1 step, 10 min	27.5 ± 6.6%	D-amino acid analogue of WT-pHLIP azide	Cu-Acetate/NaAsc	85 min	5–20%	[61]

**propargyl 4-** **[^18^F]** **fluorobenzoate** **([^18^F]** **PFB)**	Precursor: 2 steps, labeling: 1 steps, 15 min	58 ± 31%	Benzyl azide, two lysine derivatives, transglutaminase-reactive peptide	CuSO_4_/Asc	1 h (estimated)	88 ± 4%, 79 ± 33% and 75 ± 5% 37 ± 31%	[62]
4-[^18^F]fluoro-3-nitro-N-2-propyn-1-yl-benzamide ([^18^F] FNPB)	1 step, 40 min	58%	Azido-peptides cRGDfK and D4 peptide		1 h	87–93%	[63]

**1-(azidomethyl)-4-** **[^18^F]** **-fluorobenzene**	4 steps, 75 min	34%	4-Ethynyl-*L*-phenylalanine-peptide	CuI/NaAsc/DIEA	90 min	90%	[64]
	4 steps, 75 min	41%	siRNA alkyne	CuSO_4_/Asc/TBTA	120 min	15 ± 5%	[65]
	1 step, 45 min	84%	siRNA-linker (two new alkyne-bearing linkers)	CuSO_4_/Asc	120 min	12%	[66]
1-Azido-4-(3-[^18^F] fluoropropoxy)benzene	4 steps, 75 min	35%			120 min	15 ± 5%	[65]
[^18^F] (azidomethyl)fluorobenzene	1 step, 94–188 s	around 40%	siRNA alkyne		n.d.	n.d.	[67]
4-[^18^F]Fluorophenylazide		around 15%					

**3,4,6-tri-O-acetyl-2-deoxy-2-** **[^18^F]** **fluorogluco-pyranosyl azide**	1 step, 30 min	71 ± 10%	Fmoc-L-propargylglycine	CuSO_4_/Asc	1.5 h (estimated)	60%	[68]
	2 step, 7.5 min	n.d.	Alkyne-functionalized peptides (RDG, neurotensin peptoid)		75 min	17–20% n.d.c.	[69]
		52%	folate alkyne	Cu-Acetate/NaAsc	3 h	5–25%	[70]
	1 step, 10 min	84%	RGD-peptide alkyne	CuSO_4_/Asc	70–75 min	16–24%	[71]
	1 step	1.3–4.7%	Alkyne-bearing protein	CuBr/TTMA	80–100 min	4.1%	[72]
		n.d.	ET_A_R ligand alkyne	CuSO_4_/Asc	70 min	20–25% n.d.c.	[73]
			cyanoquinoline (EGFR) alkyne		90 min	8.6 ± 2.3% n.d.c.	[74]

**[^18^F]** **A** **r** **B** **F**_3_^−^	1 step, 20 min	n.d.	Alkyne-functionalized RGD	Cu^I^/Asc	1 h	n.d.	[75]
			Alkyne-functionalized bombesin (BBN)			20 ± 10% n.d.c.	[76]
	2 steps,		Alkyne-functionalized RGD-boronate		30 min	15–30%	[77]

**piperazine-based ** **[^18^F]** **AFP** **[^18^F]** **BFP**	AFP: 4 steps, 54 h BFP: 4 steps, 72 h [^18^F]AFP: 1 step, 40 min [^18^F]BFP: 1 step, 40 min	[^18^F]AFP: 29 ± 5% [^18^F]BFP: 31 ± 9%	N-Fmoc-e-azido-Lnorleucine (amino acid), SNEW peptide	CuSO_4_, Asc	2 h	Amino acid: 59–79% SNEW peptide: 17–25%	[78]

**[^18^F]** **serine**	2 steps, 125 min	28 ± 5%	cRDG-azide	CuSO_4_, Asc	145 min	75%	[6]

^1^Calculated as sum from all steps, for the ^18^F-prosthetic group, respectively, for the overall reaction yielding the click product, starting from fluorine-18.

^2^Radiochemical yields for the ^18^F-prosthetic group starting from fluorine-18 for the click reaction, respectively; decay corrected, as long as not noted elsewise.

CCA: click cycloaddition; (n.)d.c.: (not) decay corrected; Asc: ascorbate; DIPEA: diisopropylethylamin; TBTA: tris[(1-benzyl-1*H*-1,2,3-triazol-4-yl)methyl]amine; n.d.: no data.

**Table 2:** Summary of the prosthetic groups, reaction conditions, and reaction partners applied for copper-free click fluorination.

^ 18^F-prosthetic group	Steps/reaction time^1^	RCY^2^	Reacting agent	Reaction type/catalytic system	Overall reaction time^1^ (CCA)	RCY^2^ CCA	Literature
[^18^F]COT	1 step, 15 min	71%	3,6-diaryl-*s*-tetrazine	inverse electron-demand DA cyclo-addition	30 min (without HPLC)	>98%	[14]

[^18^F]FB-DBCO	1 step, 60 min	85%	Various azides	Strain-promoted click 1,3-dipolar cycloaddition	2 h	69–98%	[7]
TCO-derivative: Aza-DBCO-BN (bombesin)	9 steps, —	17%	Three different [^18^F]azides		30 min (without HPLC)	19–37% (depending on azide)	[8]
[^18^F]DBCO	1 step, 1 h	21%	Tyr^3^-octreotide-N_3_(TATE)		1.5 h	95%	[9]

[^18^F]TCO	[14]	[14]	Tetrazine-RGD	Inverse electron-demand DA cyclo-addition	30 min	90%	[15]

[^18^F]bifunctional azadibenzocyclo-octyne	1 step, 30 min	24.5%	Alkyl azide	Strain-promoted click 1,3-dipolar cycloaddition	202 ± 34 min	74 ± 4.8%	[10]
[^18^F]PEG_4_ azide	1 step, 45 min	63%	cRGD-DBCO		80 min	92%	[11]
[^18^F]cyclooctyne	6–11 steps, 30–80 h (depending on the derivative)	20–57% (depending on the derivative)	[^18^F]2-fluoro- ethylazide		30 min.	9.6–97% (depending on COT and solvent)	[12] [79]

[^18^F]*trans*-cyclooctene ([^18^F]TCO)	1 step, 102 min	46.1 ± 12.2%	Tetrazine modified exendin-4	Inverse electron-demand DA cycloaddition	3 h	46.7 ± 17.3%	[16]
			Polymer modified tetrazine			89.2% *in vivo *	[80]
[^18^F]amine-functionalised norbornene	1 step, 52 min	60 ± 17%	Tetrazine (peptide-/bombesin-derivatives)		82 min (without preparation of [^18^F]SFB)	46–97% (depending on the tetrazine)	[18]

[^18^F]FBA-C_6_-DBCO	[10]	[10]	*α*_V_ *β*_6_-specific peptide	Strain-promoted click 1,3-dipolar cycloaddition	click: 40 ± 4 min	11.9 ± 3.2%	[13]

^1^Calculated as sum from all steps, for the ^18^F-prosthetic group, respectively, for the overall reaction leading to the click product, starting from fluorine-18.

^2^Radiochemical yields for the ^18^F-prosthetic group starting from fluorine-18 for the click reaction, respectively; decay corrected, as long as not noted elsewise.

CCA: click cycloaddition; DA: Diels Alder; DBCO: *aza*-dibenzocyclooctyne; TCO: *trans*-cyclooctyne.

**Table 3:** New developments in ^18^F-click [3+2]cycloadditions, showing the 1,3-dipolar ^18^F-prosthetic groups, reaction type, and conditions.

^ 18^F-prosthetic group	Steps/reaction time	RCY	Reacting agent	Reaction type/ catalytic system	Overall reaction time^1^ (CCA)	RCY CCA	Literature
*C*-(4-[^18^F]fluoro-phenyl)-N-phenyl-nitrone	2 steps/20 min, (labeling of [^18^F]FB-CHO: 1 step, 50 min)	22–37%^1^ ([^18^F]FB-CHO: 30–50%) (^18^F-nitrone: 74%)	Various maleimides		80 min (10 min)	87–91%	[19]
4-[^18^F]fluoro-benzonitrile oxide	3 steps/20 min (labeling of [^18^F]FB-CHO: 1 step, 50 min)	28–46%^1^ ([^18^F]FB-CHO: 30–50%) (^18^F-nitro oxide: 92%)	Various dipolarophiles	1,3-dipolar [3+2]cycloaddition, no catalyst	80 min (10 min)	36–99%	[20]
			Cyclononyne-indomethacins (COX-2 inhibitor)			81%	
			Maleimide-indomethacins (COX-2 inhibitor)			55%	
			Propyne-indomethacins (COX-2 inhibitor)			35%	
*N*-hydroxy-4-[^18^F]fluorobenz-imidoyl chloride	4 steps/20 min (labeling of [^18^F]FB-CHO: 1 step, 50 min)	27–45%^1^ ([^18^F]FB-CHO: 30–50%) (^18^F-nitro oxide: 92%) (^18^F-benzimidoyl Cl: 99%)	Cyclononyne-*β*-Ala-Phe-OMe (dipeptide)		85 min (10 min)	88%^2^	
			Norbornene-*β*-Ala-Phe-OMe (dipeptide)			82%^2^	

*C*-(4-[^18^F]fluoro-phenyl)-N-phenyl-nitrone	2 steps/20 min, (labeling of [^18^F]FB-CHO: 1 step, 50 min)	22–37%^1^ ([^18^F]FB-CHO: 30–50%) (^18^F-nitrone: 74%)	Terminal alkynes methyl propiolate	*radio*-Kinugasa, CuSO_4_, AscONa (*L*-histidine)	80 min (10 min)	89% (*trans/cis* = 2 : 3)	[21]
			Terminal alkyne propargyl alcohol	*radio*-Kinugasa, CuI (Cu^I^-stabilizing ligands or pyridine)	100 min (30 min)	82% (*trans/cis* = 1 : 5) 60% (*trans/cis* = 1 : 5)	
			Terminal alkyne 1-propargyl uracyl (nucleobase chimera)			65% (*trans/cis* = 4 : 1)	
			propiolyl-*β*-Ala-Phe-OMe (dipeptide)	*radio*-Kinugasa, CuSO_4_, AscONa (*L*-histidine)	80 min (10 min)	85% (*trans/cis* = 1 : 3)	
			propiolated protein (BSA)			32%	
*o*-/*p*-[^18^F]fluoro-phenyl acetylene	n.d.	n.d.	3,6-dihydro-2*H*-1,4-oxazine-4-oxide	*radio*-Kinugasa, CuI (1,10-phenanthroline)	(10 min)	52% (*ortho*) 41% (*para*)	

^1^Calculated as sum from all steps.

^2^Best RCY, obtained only with high precursor amounts.

FB-CHO: 4-fluorobenzaldehyde; CCA: click cycloaddition; PHA: *N*-phenylhydroxylamine; AscONa: sodium ascorbate'; BSA: bovine serum albumin; n.d.: no data.
